# Supplementary figures and images for: Dinoflagellate Phosphopantetheinyl Transferase (PPTase) and Thiolation Domain Interactions Characterized Using a Modified Indigoidine Synthesizing Reporter
Source: Microorganisms. 2022 Mar 23;10(4):687. doi: 10.3390/microorganisms10040687 (PMC9027781; doi:10.3390/microorganisms10040687)

# PCPS Indigoidine Production

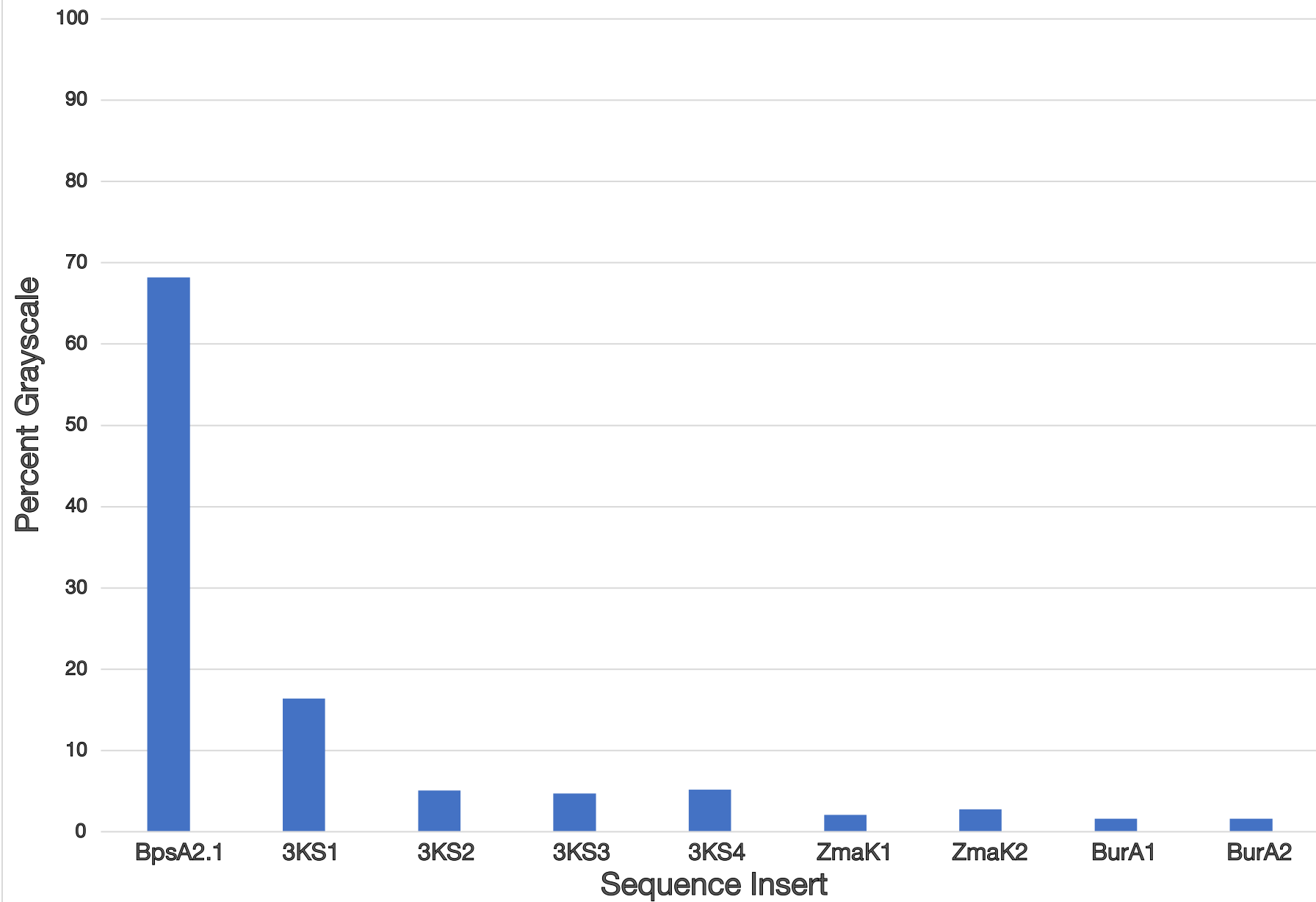

Supplement: Supplementary file 1 [file microorganisms-10-00687-s001.zip › microorganisms-1585421-supplementary.pdf]
